# Metagenome of the gut of a malnourished child

**DOI:** 10.1186/1757-4749-3-7

**Published:** 2011-05-20

**Authors:** Sourav Sen Gupta, Monzoorul Haque Mohammed, Tarini Shankar Ghosh, Suman Kanungo, Gopinath Balakrish Nair, Sharmila S Mande

**Affiliations:** 1 National Institute of Cholera and Enteric Diseases, Kolkata, India; 2 Bio-Sciences R&D Division, TCS Innovation Labs Hyderabad, Tata Consultancy Services Limited, Hyderabad, India

## Abstract

**Background:**

Malnutrition, a major health problem, affects a significant proportion of preschool children in developing countries. The devastating consequences of malnutrition include diarrhoea, malabsorption, increased intestinal permeability, suboptimal immune response, etc. Nutritional interventions and dietary solutions have not been effective for treatment of malnutrition till date. Metagenomic procedures allow one to access the complex cross-talk between the gut and its microbial flora and understand how a different community composition affects various states of human health. In this study, a metagenomic approach was employed for analysing the differences between gut microbial communities obtained from a malnourished and an apparently healthy child.

**Results:**

Our results indicate that the malnourished child gut has an abundance of enteric pathogens which are known to cause intestinal inflammation resulting in malabsorption of nutrients. We also identified a few functional sub-systems from these pathogens, which probably impact the overall metabolic capabilities of the malnourished child gut.

**Conclusion:**

The present study comprehensively characterizes the microbial community resident in the gut of a malnourished child. This study has attempted to extend the understanding of the basis of malnutrition beyond nutrition deprivation.

## Background

Malnutrition is a major global problem. While one form of malnutrition (referred to as under-nutrition) encompasses stunting, wasting, and deficiencies of essential vitamins and minerals, the other form manifests as obesity due to over-consumption of specific nutrients. The prevalence of underweight, stunting, and wasting in children, the most reliable measures of malnutrition, is concentrated in few countries in South Asia and Eastern Africa [1,2] where 33% and 28%, respectively, of the children younger than 5 years are underweight. Despite its rapid economic growth in the last decade, the estimated prevalence of child stunting in India is 51%. This translates to approximately 61 million stunted children, constituting 34% of the global total. Efforts to reduce the proportion of underweight children by half by the year 2015 is set as the first millennium development goal (MDG-1) by the United Nations.

The consequences of malnutrition are devastating. These include diarrhoea, malabsorption, small bowel overgrowth, increased intestinal permeability, enteropathy, gram-negative (enteric) bacteraemia, and suboptimal immune response [3,4]. Nutritional interventions and dietary solutions have not been the most effective till date for treatment of malnutrition. However, recent literature sheds some light on our simplistic understanding of the basis of malnutrition. Composition of host gut microbiome has been thought to play an extremely important role in absorption of nutrients from food and response to caloric deficit. Equal access to calories is now thought to be not limiting in the establishment of malnutrition. Even enteric infections result in malabsorption of nutrients due to intestinal inflammation and contribute to malnutrition in large proportions of children in developing countries. The genomes of large number of microbes present in the human gut endow us with physiological capabilities that we have not had to evolve on our own and contribute immensely in manifestation of who we are genetically and metabolically, and a reflection of our state of well being. Recent development of metagenomic procedures has now enabled us to access the complex cross-talk between the gut and its microbial flora and understand how a different community composition affects various states of human health.

In the present study, we examined the gut microbiota using faecal samples from a malnourished child and another apparently healthy child as a control from a typical urban slum setting in Kolkata, India and attempted to identify compositional and functional differences as derived from the two metagenomes. We anticipated that by analysing the gut microbial communities from the two subjects, we could have an understanding of the underlying influence of bacterial inhabitants of the gut in malnourished and healthy conditions. The overall objective was to understand the relationship between the nutritional status and the microbial community in the gut.

## Results and Discussion

Pyrosequencing of metagenomes obtained from malnourished and healthy child faecal DNA samples yielded 14,96,170 and 12,71,252 high-quality sequence reads, respectively. Eu-Detect analysis (http://metagenomics.atc.tcs.com/Eu-Detect/) followed by subsequent BLASTn searches against the human genome identified 10.3% of the sequences in the malnourished and 0.3% sequences in the healthy child data sets to have originated from human DNA. This indicates a probable ex-foliation of human tissues, manifested as contamination in the sequenced faecal sample obtained from the malnourished child.

**Figure 1:**
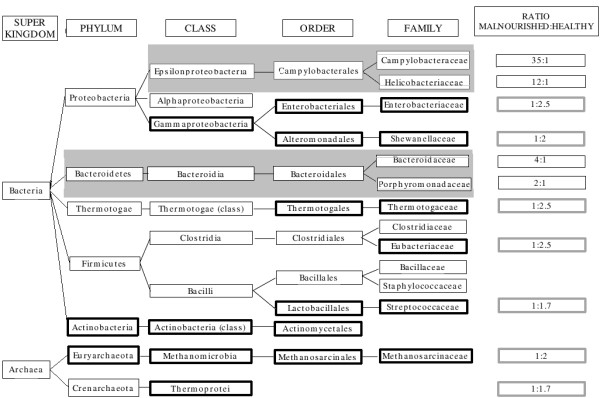
**Schematic diagram indicating taxa/lineages overabundant in malnourished and healthy child gut data sets**. Area shaded in gray: Lineages observed to be overabundant in the malnourished child sampleBold boxes: Taxa abundant in the healthy child sample

A total of 41.1% and 37% of reads in malnourished and healthy child data sets, respectively, could be classified under various taxonomic groups using SPHINX algorithm [5]. Results indicated a striking abundance of four bacterial lineages in the gut of the malnourished child as compared to the healthy child (Figure 1, Table 1).

**Table 1:** Comparison of the taxonomic assignments obtained (using SPHINX algorithm) for malnourished and healthy samples at the taxonomic levels of family, order, class and phylum.

		% of sequences assigned			
				
Taxonomic Level	Taxon Name	Malnourished Sample (X)	Healthy Sample (Y)	Relative Ratio (X/Y)	Inference
	**Campylobacteraceae**	**16.7**	**0.5**	**35.3**	**High in malnourished**
	**Helicobacteraceae**	**12**	**1**	**12**	**High in malnourished**
	**Bacteroidaceae**	**12.8**	**3**	**4.2**	**High in malnourished**
	**Porphyromonadaceae**	**5**	**2.8**	**1.7**	**High in malnourished**
	Clostridiaceae	5.9	4	1.5	-
	Bacillaceae	3.7	4.9	0.8	-
Family	Staphylococcaceae	1.3	2.1	0.6	-
	**Streptococcaceae**	**6.1**	**10.4**	**0.6**	**High in healthy**
	**Enterobacteriaceae**	**12.2**	**22.5**	**0.5**	**High in healthy**
	**Methanosarcinaceae**	**1**	**2.2**	**0.5**	**High in healthy**
	**Thermotogaceae**	**1.1**	**2.6**	**0.4**	**High in healthy**
	**Shewanellaceae**	**1.6**	**4.1**	**0.4**	**High in healthy**
	**Eubacteriaceae**	**1.9**	**5.1**	**0.4**	**High in healthy**

	**Campylobacterales**	**26.64**	**1.32**	**20.25**	**High in malnourished**
	**Bacteroidales**	**17.42**	**6.33**	**2.75**	**High in malnourished**
	Clostridiales	7.09	8.36	0.85	-
	Bacillales	10.09	12.84	0.79	-
Order	**Lactobacillales**	**6.13**	**10.39**	**0.59**	**High in healthy**
	**Enterobacteriales**	**10.71**	**19.09**	**0.56**	**High in healthy**
	**Methanosarcinales**	**1.43**	**2.63**	**0.55**	**High in healthy**
	**Alteromonadales**	**1.98**	**4.3**	**0.46**	**High in healthy**
	**Thermotogales**	**0.89**	**2.08**	**0.43**	**High in healthy**
	**Actinomycetales**	**0.54**	**2.2**	**0.25**	**High in healthy**

	**Epsilonproteobacteria**	**21.99**	**1.06**	**20.79**	**High in malnourished**
	**Bacteroidia**	**14.25**	**4.51**	**3.16**	**High in malnourished**
	Clostridia	6.46	6.6	0.98	-
	Bacilli	16.77	20.49	0.82	-
Class	Alphaproteobacteria	1.75	2.43	0.72	-
	Methanomicrobia	1.88	2.96	0.64	-
	**Gammaproteobacteria**	**24.28**	**41.37**	**0.59**	**High in healthy**
	**Thermoprotei**	**1.21**	**2.09**	**0.58**	**High in healthy**
	**Actinobacteria (class)**	**0.61**	**2.49**	**0.24**	**High in healthy**

	**Bacteroidetes**	**13.95**	**4.88**	**2.86**	**High in malnourished**
Phylum	Proteobacteria	49.78	50.61	0.98	-
	Firmicutes	24.28	26.27	0.92	-
	**Euryarchaeota**	**3.95**	**7.03**	**0.56**	**High in healthy**

**Note**: 1. Only those taxa which had at least 2% of the sequences assigned to it (in either samples) were considered for comparison

2. For each taxa, the relative ratio was obtained by dividing the percentage of a taxon observed in the malnourished sample by the percentage of the same taxon in the healthy sample.

3. Those taxa having a relative ratio of more than 1.5 or not present in the healthy sample were tagged as 'High in malnourished', while those taxa which had a relative ratio of less than 0.6 were tagged as 'High in healthy'.

Families Campylobacteraceae and Helicobacteraceae were 35 and 12 folds higher in the malnourished child sample (Figure 1) suggesting infection of the intestinal epithelium by gastrointestinal pathogens belonging to these families. It is well known that infection adversely affects nutritional status and also that malnutrition can predispose to infection [6]. Though it is difficult to predict the exact sequence of events in the vicious cycle of infection and malnutrition, the present study underscores the presence of pathogens normally absent in the intestines of healthy subjects. Species belonging to Campylobacter and Helicobacter are well established human pathogens. For example, *Campylobacter jejuni *and *Campylobacter coli *are known to be the causative agents of Campylobacteriosis, a collective description for diarrhoeal disease caused by members of the Campylobacter genus. Similarly, the microaerophilic bacterium Helicobacter, known to inhabit various areas of the stomach (particularly the antrum), causes a chronic low-level inflammation of the stomach lining and is linked to the development of duodenal and gastric ulcers and stomach cancer. An association between reduction in gastric acid and *Helicobacter pylori *infections has been linked to increase in *Vibrio cholerae *infections [7,8]. Individuals with gastric hypochlorhydria or achlorhydria are at greater risk of developing cholera after infection with low inoculums [9]. The high density of members of the family Helicobacteraceae in the malnourished child gut metagenome would predispose this child to repeated infections because gastric acid is known to act as a natural non specific barrier to enteric pathogens. Therefore, in the malnourished children, a lower magnitude of bacterial inoculum would give rise to severe infection as compared to their healthy counterparts. In the healthy child gut metagenome, such a predilection of Helicobacteraceae sequences was not observed suggesting that the healthy flora could competitively inhibit and exclude extraneous pathogens. A malnourished child, in contrast, would suffer from repeated bouts of infections (and disease) and this coupled with poor nutrient absorption would drive the child into throes of declining health and ultimately to death.

Bacteroidaceae family was also observed to have an appreciably higher representation in the malnourished sample (Figure 1). In contrast to 3% of sequences in the healthy child sample, approximately 13% of sequences belonged to this family in the malnourished child sample. An increase in abundance of this bacterial phylotype has been previously associated with a decrease in body weight of obese individuals [10]. Similarly, the family Porphyromonadaceae was also seen to have approximately a two-fold enrichment in the malnourished child sample. Interestingly, members of the Porphyromonadaceae family were shown to be exclusively present in the faecal microbiota of patients suffering from Crohn's disease, an inflammatory bowel disease [11].

**Figure 2:**
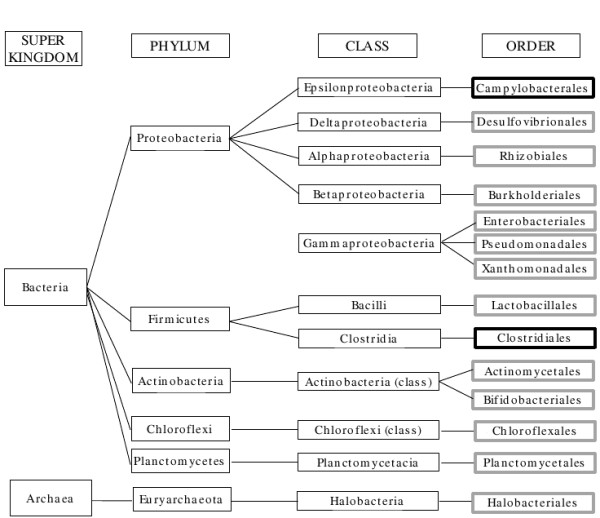
**Schematic diagram showing the taxonomic distribution of sequences identified as specific to malnourished and healthy child gut data sets**. Bold black boxes: Taxa associated with sequences identified as specific to malnourished child data setBold grey boxes: Taxa associated with sequences identified as specific to healthy child data set

To understand the functional differences between these two gut metagenomes, we used HabiSign algorithm (http://metagenomics.atc.tcs.com/HabiSign/) to identify sequences unique to either healthy or malnourished samples. The taxonomic affiliations of these sequences were inferred before functionally analysing them. 79% of the sequences specific to the malnourished child sample belonged to the order Campylobacterales (Figure 2, Table 2). Another subset of malnourished sample specific sequences belonged to the order Clostridiales. Surprisingly, this order was observed to be equally represented in both samples by the SPHINX-based analysis (Table 1). This observation indicates the presence of novel species belonging to the order Clostridiales in the malnourished child metagenome.

**Table 2:** Comparison of the taxonomic assignments obtained (using SPHINX algorithm) for sequences specific to malnourished and healthy samples (at the taxonomic level of order)

	% of sequences assigned			
			
Order Name	Malnourished sample (X)	Healthy Sample (Y)	Relative Ratio (X/Y)	Inference
Campylobacterales	78.87	0.06	1235.57	High in malnourished
Clostridiales	11.4	0.1	119.1	High in malnourished
Lactobacillales	0.79	3.19	0.25	High in healthy
Enterobacteriales	0.11	5.07	0.02	High in healthy
Pseudomonadales	0.04	5.27	0.01	High in healthy
Chloroflexales	0.02	4.44	0	High in healthy
Desulfurovibrionales	0	4.44	0	High in healthy
Bifidobacteriales	0	6.98	N.A	High in healthy
Xanthomonadales	0	3.21	N.A	High in healthy
Plantomycetales	0	2.13	N.A	High in healthy
Halobacteriales	0	12.24	N.A	High in healthy
Actinomycetales	0	19.72	N.A	High in healthy
Burkholderiales	0	19.39	N.A	High in healthy
Rhizobiales	0	2.39	N.A	High in healthy

In contrast, sequences specific to the healthy child data set were seen to be enriched for the following orders - Lactobacillales, Enterobacteriales, Pseudomonadales, Chloroflexales, Xanthomonadales, Planctomycetales, Halobacteriales, Burkholderiales, Actinomycetales, Bifidobacteriales, Desulfovibrionales and Rhizobiales (Figure 2, Table 2). The microbial fitness of the healthy child gut in performing normal gut function is enhanced due to the overall abundance of these bacterial phylotypes and absence of potentially harmful enteropathogens. A thriving gut bacterial community in the healthy child also helps in the proper maturation of immunity which in turn aids in defence against gut pathogens. As a result of reduced abundance of many beneficial members of the known bacterial phylotypes in the malnourished gut as compared to the healthy child, there is also a reduced availability of usable microbial fermented products from otherwise indigestible dietary polysaccharides and microbe derived nutrients in the malnourished gut.

**Figure 3:**
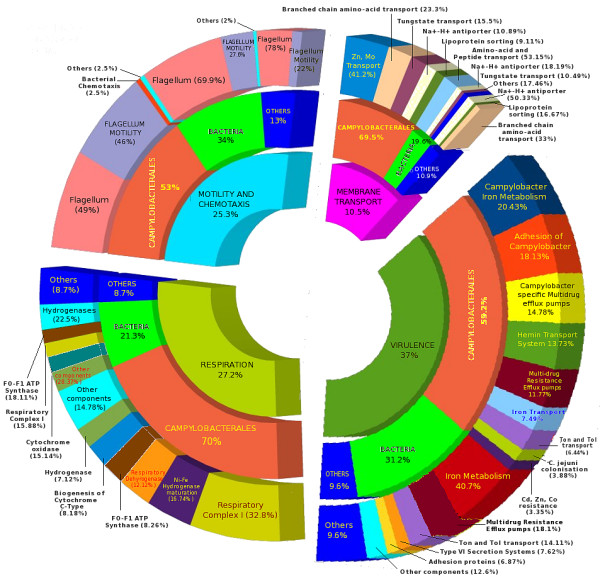
**Pi chart illustrating taxonomic and functional characterization (subsystems and associated PEGs) of the malnourished-specific sequences**. Innermost layer: Identified subsystem categoriesMiddle layer: Taxonomic mapping of specific sequences within each categoryOuter layer: PEGs associated with each identified subsystem

Analysis of the results obtained from the Meta Genome Rapid Annotation using Subsystem Technology (MG-RAST) server [12] for the identified malnourished/healthy metagenome specific sequences indicated the presence of functionally characterized protein encoding genes (PEGs) that were specifically abundant in either sample (Table 3). Results indicated that PEGs belonging to four subsystems (motility and chemotaxis, respiration, membrane transport, virulence) were relatively more abundant in sequences identified as specific to the malnourished child sample (Figure 3).

**Table 3:** Functional analysis of sample (malnourished/healthy) specific sequences

Subsystem category	% of Malnourished specific sequences (X)	% of Healthy specific sequences (Y)	Relative Ratio (X/Y)	Relative Ratio (Y/X)
Motility and Chemotaxis	4.18	0.04	104.5	0.01

Membrane Transport	2.12	0.21	10.1	0.1

Respiration	5.37	1.28	4.2	0.24

Virulence	9.49	5.28	1.8	0.56

Stress Response	1.95	1.83	1.07	0.94

Protein Metabolism	12.1	11.44	1.06	0.95

Amino Acids and Derivatives	8.45	8.54	0.99	1.01

DNA Metabolism	7.01	7.37	0.95	1.05

Cofactors, Vitamins, Prosthetic groups and pigments	7.07	7.71	0.92	1.09

Cell wall and Capsule	6.52	7.38	0.88	1.13

Carbohydrates	5.78	7.95	0.73	1.38

Clustering-based Subsystems	11.5	16.24	0.71	1.41

RNA Metabolism	5.11	7.58	0.67	1.48

Cell Division and Cell Cycle	1.61	2.43	0.66	1.51

Unclassified	2.26	4.45	0.51	1.97

Nucleosides and Nucleotides	3.38	6.66	0.51	1.97

**Note**: Only those subsystem categories which had at least 1.5% of the sequences assigned to it were considered for comparison

Flagellum, flagellum motility subsystems and motility accessory proteins, all mapping to species belonging to the order Campylobacterales, were abundant in the malnourished child sample under the motility and chemotaxis subsystem (Figure 3). Similarly, approximately 70% of malnourished specific sequences functionally classified under the 'respiration' category were also seen to be taxonomically assigned to the order Campylobacterales (Figure 3).

Malnourished specific sequences tagged to the 'membrane transport' subsystem also indicated an abundance (~70%) of sequences classified under the order Campylobacterales (Figure 3). While some of these sequences mapped to ABC transporters of branched-chain amino acids, Zinc, Tungstate and Molybdenum, others mapped to specialized membrane transport systems like Na(+)-H(+) antiporter and the lipoprotein sorting systems. Specific Tungstate and Molybdenum transport systems are known to exist in pathogenic Campylobacter species [13,14]. Lipoproteins are known to play a major role in the virulence of several pathogenic organisms. Besides having key roles in functional pathways like motility, chemotaxis, cell-cell interactions, and signal transduction, lipoproteins are also implicated in the assembly and regulation of bacterial secretion systems [15].

Many malnourished child specific virulence-associated sequences belonged to order Campylobacterales (Figure 3). These sequences mapped to PEGs corresponding to iron metabolism, adhesion, multidrug resistance efflux pumps, hemin transport system, iron transport, Ton and Tol systems. 31% of virulence-associated malnourished specific sequences, mapping to the above PEGs, could only be assigned at the superkingdom level of Bacteria (Figure 3), indicating presence of hitherto unknown organism(s) with pathogenic potential in the malnourished child gut. Interestingly, 7.6% of the virulence-associated sequences mapped to the PEGs associated with various components (IcmF, ImpB, ImpC, ImpG, ImpH and ImpJ) of Type VI secretion system. IcmF component is similar to VasK, a protein known to play a key role in cell surface recognition and adherence of bacterial pathogens to host cells [16,17]. ImpB and ImpG have been shown to be similar to *V. cholerae *secretion systems components, namely VCA0107 and VCA0111 respectively.

The analysis of malnourished specific PEGs classified under the various subsystem categories reveals a comprehensive set of PEGs belonging to interlinked pathways or subsystems that may work together and play a critical role in contributing to the pathogenicity of Campylobacter species in the gut of the malnourished child.

## Conclusion

**Figure 4:**
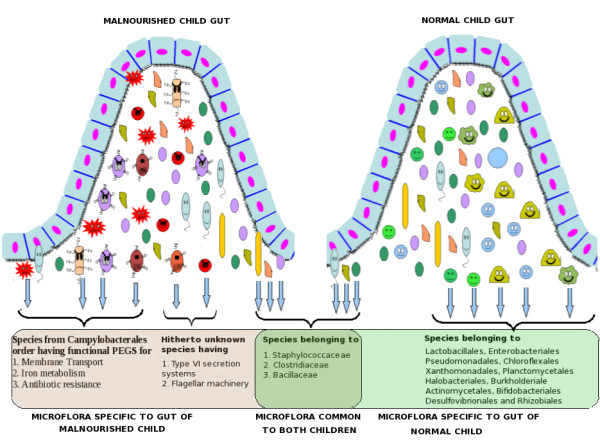
**Schematic diagram indicating the overall differences between microbial communities residing in the gut of a malnourished and a healthy child**.

This study reveals a model of the gut microbiome of a malnourished child residing in an urban slum setting in Kolkata where children are constantly exposed to enteric pathogens because of poor sanitation and hygiene and due to consumption of contaminated drinking water. Life expectancy in such resource limited setting is intriguingly dependent on physiological mechanisms of the child to prevent the swarm of extraneous pathogens. The overall differences between microbial communities residing in the gut of the malnourished and healthy child are illustrated in Figure 4. The intestinal microflora of the malnourished child when compared to the healthy child is interpreted as aberrant gut microflora. Such an aberration leads to a sub-clinical disorder characterized by inflammation and modest malabsorption. The sequel of events following the continued aberration of the intestinal microflora includes unchecked bacterial proliferation, concurrent infection, disruption in community dynamics of commensal intestinal flora and impaired immunity. Each of these events would exacerbate the other. The further reduction in diversity of essential bacterial phylotypes in the gut would result in declining gut function and competition for nutrients resulting in the downward spiraling of the child's health. Maintenance of a healthy gut microflora as observed in the healthy child included in this study therefore seems to ensure the ability to exclude pathogens and perform its normal functioning. Apart from the current focus on dietary solutions, prevention, treatment and management of malnutrition should aim to protect the normal gut flora from infection by enteric pathogens and allochthonous microflora. An interesting question is 'Despite living under similar conditions of hygiene and sanitation, why does a child become malnourished while the other remains normal?'. To completely understand the role of gut microbiota in malnutrition in millions of children across the globe, other models from different socio-economic backgrounds, geographically distinct locations and diverse age groups need to be investigated.

## Methods

### Samples and DNA extraction

Faecal samples were collected aseptically in sterile stool containers from a healthy and a severely malnourished infant. Both the infants were 16 month old females. Samples were transported to the laboratory using frozen ice packs and were immediately stored at -80°C in 200 mg aliquots until further processing. It was ensured that no antibiotics were administered to the children for at least 3 months prior to sample collection. DNA was extracted using the bead beating method and subsequently using the Qiagen Stool DNA Mini kit. Qiagen stool lysis buffer was added to the frozen samples and used for whole community DNA extraction. 300 mg of 0.1 mm zirconia/silica beads (BioSpec Products) were added to each tube and microbial cells were then lysed by mechanical disruption with a bead beater (BioSpec Products) set on high for 2 mins. The DNA was precipitated using ethanol after removal of the inhibitors using the Qiagen InhibitEX tablets and treatment with proteinase K. DNA was purified by binding on a QIAamp mini spin column and subsequent washing with wash buffers and finally eluted in 200 μl TE (pH 8.0). The quantity and quality of purified DNA was assessed spectrophotometrically and also by using agarose gel electrophoresis.

### Community Metagenome Sequencing

Total community DNA extracted from each stool specimen was directly sequenced on individual pyrosequencing slides on a ROCHE 454 GS FLX (Roche Diagnostics, Inc. Basel, Switzerland) sequencing instrument. While the healthy child sample was sequenced using the GS FLX chemistry, the malnourished child sample was sequenced using the Titanium chemistry. The distinct difference in read lengths in these two methods did not affect the downstream bioinformatic processes as we undertook an assembly independent analysis of the two metagenomes.

### Bioinformatic analysis

Low quality and short sequences were first removed from both data sets using in-house scripts. Duplicated sequences, a known artifact in pyrosequencing data, were also removed. Using Eu-Detect algorithm (http://metagenomics.atc.tcs.com/Eu-Detect), sequences of probable eukaryotic origin were identified. Sequences originating from human DNA were then identified by performing a BLASTn search of these sequences against human genome sequences. Sequences having at least 80% identity (spread over at least 80% of the length) to human sequences were removed from subsequent analyses.

All reads in both data sets were taxonomically classified using the SPHINX algorithm [5]. Analysis of these assignments at various taxonomic levels was performed by first collapsing all assignments at a desired taxonomic level, and subsequently enumerating the number of assignments to various taxa at that level.

Sequences specific to either the malnourished or the healthy child data set were identified using the HabiSign algorithm (TSG *et al*., manuscript communicated). This algorithm identifies sequences specific to a data set by first mapping all sequences to pre-computed points in feature vector space. Subsequently, sequences specific to a data set are identified by finding regions in the same feature vector space that are observed to be selectively over-mapped by sequences belonging to that data set. For each data set, the taxonomic affiliations of the identified specific sequences were inferred from the results of SPHINX analysis.

Functional categorization of the sequences identified as specific to each data set were performed by submitting these sequences to MG-RAST server (http://metagenomics.nmpdr.org/). The SEED platform hosted on this server contains all protein sequences classified under various sub-systems [12]. The specific sequences were compared against the proteins in various sub-systems using BLASTx with an e-value cutoff of e-10, percent identity greater than 66% and an alignment length of greater than 50 bases. The percentage of specific sequences tagged to various functional categories were obtained and subsequently compared.

## Abbreviations

MDG-1: First Millennium Development Goal; MG-RAST: Metagenome Rapid Annotation using Subsystems Technology; PEGs: Protein Encoding Genes

## Competing interests

The authors declare that they have no competing interests.

## Authors' contributions

SSG prepared DNA from the faecal samples and collated the information on the sequences, SK collected the samples and information for categorizing the child as malnourished, MHM, TSG and SSM performed detailed bioinformatics analysis. SSG, MHM, TSG, GBN and SSM analyzed the results and wrote the paper. All authors read and approved the final manuscript.

## Consent

Written informed consent was obtained from the parents of the two children for publication of this case report and accompanying images. A copy of the written consent is available for review by the Editor-in-Chief of this journal.
